# Supplementary material for: Chromothripsis during telomere crisis is independent of NHEJ, and consistent with a replicative origin
Source: Genome Res. 2019 May;29(5):737–49. doi: 10.1101/gr.240705.118 (PMC6499312; doi:10.1101/gr.240705.118)
Supplement: Supplemental Material [file supp_gr.240705.118_Supplemental_file_1.zip › contigs/annotated_contigs/DB115/contig.2.DB115_length_619_mean_cov_9.88045234249.docx]

**DB115_length_619_mean_cov_9.88045234249**

```
TAATGACGTACTGACATGTACTACCACATGGATGAATCTCTAATGCATTGACCCAAAAACCTACCGTATGATTCAATTTACGTGACATG
 >chr2:163017019-163017339 - E=7e-181
TTCAGAACACGTAAATCCATAGAGACAGAGTTCAAAGTAGTGGTTGCCACTGGGTGTGGGGAGAGAGATGTGAGGAGAAACTTCTTACT

GAATATGGGTTTTTATTTTGAAATTATGGAATTGTTTTGGTACCTAATAGAGGTGGTGGTTGCACACATAAATGTACTAAATGTCACTT

AATTGTTCACTTTAAAAGGATTTATTTTATTATGTAAATGTCACCTCAATAAT|AAA|ATATCCAAAATTCGTGGATCTGATTCTTAAA
                                                         >chr2:163016050-163016346 - E=
AAACAGAATCTCCACTGTCGCTTTGGAAAGCAGTTGGCAGTTCCTCAAAAAGCTAAACATAGAGTTAGCATATAAGCCAGCGATTCTAC
2e-163
TCCTAGGTATTTACCCAAGAGAATTAAAAACATAGTCCAGACAAAAATTTGTGCATGAATGTTCCCAGCAGCATTATTCATAATGGCCC

CAAAGTGGAAACCACCCAGATGTCCATTACTGATAGATGGGTAAACTAGATGTGGTAAATAGCATACAATGGAATCATTTCAACCGT
```
